# A Low Complexity Persistent Reconnaissance Algorithm for FANET

**DOI:** 10.3390/s22239526

**Published:** 2022-12-06

**Authors:** Yuan Guo, Hongying Tang, Ronghua Qin

**Affiliations:** 1 Science and Technology on Micro-System Laboratory, Shanghai Institute of Microsystem and Information Technology, Chinese Academy of Sciences, Shanghai 200050, China; 2 The School of Electronic, Electrical and Communication Engineering, University of Chinese Academy of Sciences, Beijing 100049, China

**Keywords:** unmanned aerial vehicles, FANET, PSO-based, relay node placement, persistent reconnaissance, dynamic threat avoidance, low complexity

## Abstract

In recent years, with the rapid progress of unmanned aerial vehicle (UAV) technology, UAV-based systems have been widely used in both civilian and military applications. Researchers have proposed various network architectures and routing protocols to address the network connectivity problems associated with the high mobility of UAVs, and have achieved considerable results in a flying ad hoc network (FANET). Although scholars have noted various threats to UAVs in practical applications, such as local magnetic field variation, acoustic interference, and radio signal hijacking, few studies have taken into account the dynamic nature of these threat factors. Moreover, the UAVs’ high mobility combined with dynamic threats makes it more challenging to ensure connectivity while adapting to ever-changing scenarios. In this context, this paper introduces the concept of threat probability density function (threat PDF) and proposes a particle swarm optimization (PSO)-based threat avoidance and reconnaissance FANET construction algorithm (TARFC), which enables UAVs to dynamically adapt to avoid high-risk areas while maintaining FANET connectivity. Inspired by the graph editing distance, the total edit distance (TED) is defined to describe the alterations of the FANET and threat factors over time. Based on TED, a dynamic threat avoidance and continuous reconnaissance FANET operation algorithm (TA&CRFO) is proposed to realize semi-distributed control of the network. Simulation results show that both TARFC and TA&CRFO are effective in maintaining network connectivity and avoiding threats in dynamic scenarios. The average threat value of UAVs using TARFC and TA&CRFO is reduced by 3.99~27.51% and 3.07~26.63%, respectively, compared with the PSO algorithm. In addition, with limited distributed moderation, the complexity of the TA&CRFO algorithm is only 20.08% of that of TARFC.

## 1. Introduction

Due to recent advances in technology for small unmanned aerial vehicles (UAVs), the application of a flying ad hoc network (FANET) has received a significant boost in the military, industrial, and civil sectors. Small UAVs or quadcopters often have reduced performance in order to reduce weight and cost compared to traditional UAVs that can complete their missions alone. The greatest strength of small UAVs lies in their ability to form a mission network and cooperate to complete complex tasks. As a result, a multi-UAV cooperation, or FANET, has been in the spotlight of the research community over the years. Scholars have made a detailed exploration of the FANET from different perspectives, such as routing protocols, deployment, hierarchical structure, algorithm optimization, and applications.

Various routing protocols have been proposed to optimize the performance of the FANET [1,2,3,4,5]. The authors in [1] have carefully designed the application of IEEE 802.11 MAC in the FANET. Through an exhaustive performance analysis, they have obtained some instructive conclusions. Khan et al. [2] use a specifically designed protocol for FANETs that considers the interest characteristics of FANETs, but the path to destination sometimes is not optimized and creates a closed-loop route. Considering the fast dynamic nature of nodes in FANETs, Rosati et al. propose a technique in [3] to use combined directional and Omni-directional antenna to improve routing path selection and try to minimize the Expected Connection Time (EMC) and the utility function for path selection. To accommodate the communication requirements of a heterogeneous network, Oubbati et al. [4] design an interaction possibility metric in routing protocol. In this way, the protocol improves the extension of networks and coverage of sub-networks assistance to some extent. Focusing on the UAVs’ power limitation, Kai [5] structures an energy-efficient cooperative relaying scheme to extend the network lifetime while guaranteeing the success rate.

The excellent mobility of nodes in the FANET makes localization, deployment, and timely optimization of paramount importance [6,7,8,9]. The localization of UAVs is a prerequisite for algorithms to maintain network connectivity and threat avoidance, and a well-connected network can also improve localization accuracy through collaboration [6]. In [7], UAVs are envisioned as wireless base stations. The authors first calculated the coverage probability of the downlink, then used circle packing theory to determine UAVs’ locations in 3D space to maximize the coverage area and coverage lifetime. Notably, Silva et al. [8] propose a FANET topology coordination protocol based on Software-Defined Network (SDN). By incorporating SDN into the UAV deployment strategy, the article sheds new light on FANET deployment optimization. To cope with the deterioration of the network connection caused by node vulnerability, ways to implement distributed connection maintenance and node importance assessment are extensively investigated in [9].

With the popularity of FANET technology, it has received much research interest in numerous practical applications [10,11,12,13]. To realize remote command and situational awareness, the authors in [10] constructed a cooperative monitoring network consisting of multiple UAVs and ground stations. A multi-relay UAV selection scheme based on fuzzy optimization is developed to realize the tradeoff between surveillance performance and connectivity maintenance. The articles [11,12] focus on the application of FANETs in disaster relief. In [11], Joshi et al. deal with the continuous sensing and monitoring of the geographical location of a specific disaster event. Their paper introduces and demonstrates various protocol stacks. A network simulator (NS-3) and a robot simulator (Gazebo) are used in synergy to simulate the disaster event boundary monitoring process. In addition, Sánchez-García et al. [12] propose a distributed algorithm, dPSO, to provide network support for victims and ambulance personnel in disaster areas. In the process of urban’s digital and intelligent development, FANET technology is envisaged to play an important part. Siddiqi et al. [13] designed an enhanced Ant Colony Optimization (ACO) technique for traffic detection in remote urban areas, which improves the network life and the number of received packets compared to comparison algorithms.

In the case when continuous reconnaissance is required, the detection range expansion, information transmission, and even network segmentation of a FANET remain hard nuts to crack. Moreover, the safety of UAVs cannot be guaranteed due to natural factors and various enemy air defense operations [14,15,16,17]. For example, a common natural threat stems from local magnetic field variations. Sudden magnetic field change can interfere with the magnetic compass used for UAV positioning. The UAV incorrectly assumes that the change in magnetic compass data is due to its position movement and makes corrective actions to deal with it. These actions continue with the magnetic disturbance, and the UAV is out of control as seen from the ground. Moreover, in a hostile environment, the enemy can decrypt the communication protocol of UAVs to gain control. That is a common threat that UAVs face in the process of reconnaissance. Furthermore, acoustic waves are also used to strike UAVs. When the location of a UAV is detected, the acoustic transmitter sends acoustic waves of a specific frequency to its direction, triggering a resonance effect of the UAV’s gyroscope. Once the gyroscope becomes abnormal, the UAV’s inability to identify angles could cause it to crash.

Some studies did take the above-mentioned issues into account. Zuev et al. considered the possible threats at the data transfer protocol level, such as hostile devices altering or masking the signals received by UAVs’ GPS receivers [15]. They proposed a new method to evaluate UAV security threats based on Two-Criteria Likelihood-Impact scales. In [16], electromagnetic interference is considered, and the interference suppression is realized by optimizing the airborne antenna. Taking targets’ movement into account, Song et al. [17] designed a cooperative UAV tracking method based on a sparse A* search and Standoff tracking algorithm. It realizes continuous tracking of moving targets in the task area.

However, as far as reconnaissance missions are concerned, none of the above literature considers the dynamics of the threat to drones. Nowadays, various jamming and hijacking capabilities have been integrated into diversiform mobile anti-reconnaissance devices, making the UAVs’ threat change frequently. Only when the security of UAVs is guaranteed can various network architecture schemes and collaborative tasks be executed normally.

To achieve sustained reconnaissance in hostile scenes, in this study we first proposed the Threat Avoidance and Reconnaissance FANET Construction algorithm (TARFC). Then, taking into account the movement of monitored targets and the overall changes in the hostile area, the Total Edit Distance (TED) is defined as a measure of those variations. Finally, the Dynamic Threat Avoidance and Continuous Reconnaissance FANET operation (TA&CRFO) is proposed by incorporating the TED indicator into TARFC. The algorithm reduces the complexity of TARFC by making UAVs execute adaptively and has good application value in actual scenes. The contributions of this paper are as follows:

- We introduce a constraint on the threat probability density function (threat PDF) to model the changing threats in the scene. By transforming the constrained problem into an unconstrained problem using the Lagrange Multiplier method, the PSO-based TARFC algorithm is proposed to find optimal UAV locations that stay away from threats and maintain network connectivity.
- The TED metric is put forward to measure the variation degrees of the FANET and reconnaissance scenarios over different periods of time. According to the TED value, the control center will determine whether to execute overall coordination by sending control commands or to allow each node to perform distributed adaptive adjustments based on their local information. In this way, the dependence of UAVs on the control center can be reduced.
- Combined with the above two, the TA&CRFO algorithm is designed. It can adaptively adjust the topology of the FANET in realistic scenarios and realize the dynamic continuous reconnaissance goal of the FANET with low complexity, even if the monitored targets or scenario’ threats are time-varying.

The structure of this paper is organized as follows: Section 2 mainly introduces a hierarchical architecture of the heterogeneous FANET and presents the problem-framing process. In Section 3, the PSO-based TARFC algorithm is proposed to achieve the construction of the FANET reconnaissance network and the threat avoidance in the scenario. Then, the TED metric is designed to measure the relevant changes. Finally, TA&CRFO is proposed to achieve the on-demand collaborative management of UAV nodes during continuous reconnaissance. Subsequently, Section 4 provides some analysis of simulation results, and some conclusions and future directions are described in Section 5.

## 2. System Model and Problem Statement

In this subsection, we first provide a description of the system model and then formulate an optimization problem that the TA&CRFO algorithm can handle. Table 1 and Table 2 present, respectively, the lists of acronyms and variables used in this article for the readers’ convenience.

### 2.1. System Model

In recent years, three main strategies have been used to achieve drone swarms’ persistent surveillance [8,11,12,13,18,19,20,21,22,23,24,25,26,27]: (i) on-duty UAV replacement scheme based on recharging stations [13,19,25]; (ii) energy-efficiency path planning [20,21,22,23,24,25,26,27]; and (iii) novel structures of UAV teaming [8,11,18,22]. UAV distribution’s hierarchical architecture helps plan efficient and adaptive surveillance missions when the surveillance map changes due to weather or invisible factors. The hierarchical structure intends to divide the surveillance task into different platforms such as ground stations, high-altitude UAVs, and UAV sensing swarms. Each platform is in charge of different functions such as control, motion coordination, data transmission, package routing, etc.

We assume that the FANET in this study consists of a high-altitude UAV A, as its airborne command and control platform, i.e., ACP, and set U of drone swarms in low-altitude to perform reconnaissance missions.

If there is any ambiguity, all UAVs are referred to as nodes of the network throughout this article. We identify a node i’s location by $x_{i}\in {\mathbb{R}}^{3}$, and a set of all the nodes’ locations in set V by $X_{V}={\left\{x_{i}\right\}}_{i\in V}$. To simplify the model, we presume that each UAV can only carry out one reconnaissance operation at a time and that each target that is being scouted should be covered by at least one UAV. As a consequence, we make the assumption that the number of targets to be scouted is not larger than the number of drones that are currently accessible. In our FANETs, the ACP can either hover over the given location or fly around the periphery of the area of interest, while drone swarms have controllable mobility. Therefore, drone movement can be managed by themselves or by the ACP to achieve excellent performance in both network and mission-related factors. Considering the radio propagation model, Friis’s free space model [28], the most popular propagation model, is used in this article. According to this radio propagation model, all nodes have the same transmission radius. The connection between two nodes occurs when and only when they are within the transmission radius. One of the crucial research concerns is the energy problem, which includes things such as patterns of energy consumption and battery dynamics. We direct readers to [29] and any references therein because it is outside the scope of this article.

For ease of description, we designate the UAV assigned to reconnaissance mission m as monitoring UAV (MU) and denote it as $\varepsilon_{m}$. Therefore, the set of MUs can be expressed as $M=\left\{\varepsilon_{1},\ldots ,\varepsilon_{m}\right\}$. The remaining drones that are not assigned to any reconnaissance mission are used as communication relay nodes in the network, which are in charge of transmitting data between MUs and the ACP in the upper air. Relay UAVs (RUs) is the term we use to identify them, i.e., R=U\M.

**Figure 1:**
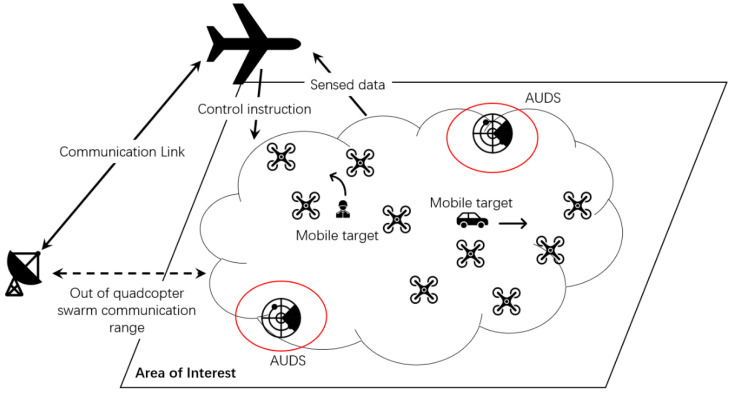
Hierarchical structure of proposed FANET persistent surveillance system.

Figure 1 describes the hierarchical structure of our UAV persistent surveillance team. Because of the quadcopter’s limited communication range, the high-altitude UAV undertakes information exchange with the remote ground end. Some necessary control instructions for drone swarms are also sent from the high-altitude UAV since its bigger role is the ACP of the network. Inside the FANET, a buffer layer of UAV swarm between the ACP and ground objects extends this system’s ability on connection service and real-time tracking. With many agents within the UAV swarm layer, an adaptive formation policy can be developed to fit various requests, including avoiding dangerous areas such as Anti-UAV Defensive System (AUDS), thunderstorm areas, strong communication interference areas, etc.

Next, it is challenging to guarantee direct communication between each UAV node and the ACP to maximize the drone swarm’s search and surveillance range. Therefore, multi-hop communication is usually adopted, which requires establishing a routing path for packet transmission. As a result, the choice of routing path significantly impacts the performance of the FANET. When coming up with a solution for the UAV locations, the routing protocol should be considered. Different UAVs may operate in different ideal locations depending on the routing protocol. Therefore, in this paper, we focus on routing protocols that offer a selection of routes between MUs and the ACP based on the positions of the nodes and presumptively employ a routing protocol that is known in advance. Accordingly, we define a routing function,

(1) $$\rho :\left\{x_{\varepsilon_{m}},x_{A},X_{R}\right\}\Leftrightarrow \rho \left(m\right),$$

where ρ(m) is the series of wireless links from the MU $\varepsilon_{m}$ to the ACP A through the relay UAVs in R.

### 2.2. Problem Formalation

Communication is the basis of cooperation and collaboration between UAVs, which is crucial and essential [10]. For simplicity, we assume that the communication between the UAVs follows the line-of-sight (LoS) model [28,30]. In the following, we introduce the concept of network connectivity and the FANET threat metric.

#### 2.2.1. Network Connectivity

We examine a network connection function that only considers active links in order to more correctly evaluate the network performance of our FANET. An active link is defined as a link that is a part of any routing path that connects the executing MU and the ACP. Due to the node location $x_{i}$’s differentiable characteristic, we define the network connectivity $f^{C}$ as the averaged value of all active links’ quality, i.e.,

(2) $$f^{C}\left(X_{V},\rho \right)=\frac{1}{\left|Ρ\right|}\sum_{\left(i,j\right)\in Ρ}f_{i,j}^{C}\left(x_{i},x_{j}\right),$$

where Ρ is the set of all active links, i.e., $Ρ=\underset{m\in M}{\cup }\rho \left(m\right)$, and $f_{i,j}^{C}$ represents the quality of the wireless link (i,j). Accordingly, we make the assumption that the $f_{i,j}^{C}$ can be defined as ${\left\|x_{i}-x_{j}\right\|}^{p}$, where ${\left\|\cdot \right\|}^{p}$ stands for the Lp-Norm. 

#### 2.2.2. FANET Threat Metric

For the sake of practical application, we define the FANET threat metric, $f^{T}$, to quantify and uniformly express various threats (military anti-reconnaissance threat, terrain features, weather conditions, communication interference, etc.) that each relay UAV faces in a scene. However, our main concern in this paper is not how to define or measure those threats posed to the UAVs by different factors but how each UAV can stay away from areas with high threat values while ensuring its reconnaissance performance and network connectivity. Therefore, in this paper, we do not discuss the modeling and quantification of the threat metric model. Instead, we give a predefined time-varying threat density distribution for the scenario.

**Figure 2:**
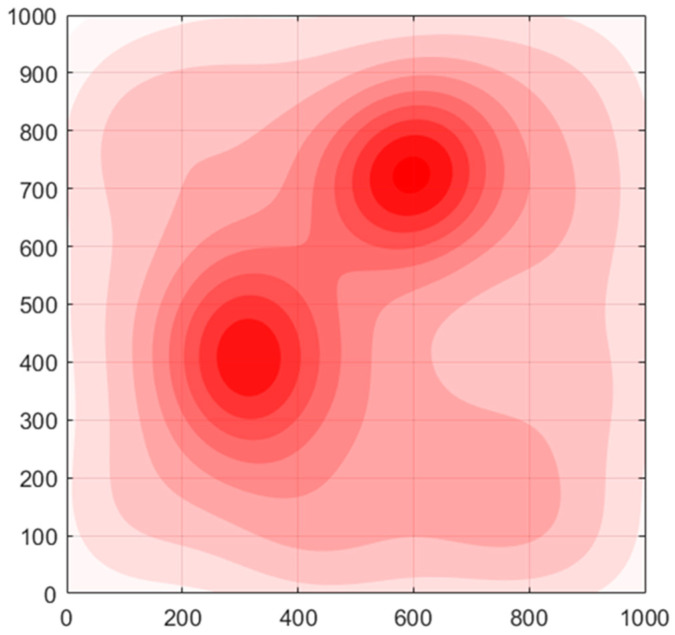
Example of threat density distribution in area of interest.

To help readers obtain a more intuitive impression, Figure 2 is used as an example to show the threat density distribution in the reconnaissance area. The threat density may come from anti-drone devices, communication jamming, etc. In the image, the darker the red, the greater the threat is. For each UAV in the simulated area, the threat value is the integral of the threat density in its associated area.

The $f^{T}$ is defined by

(3) $$f^{T}\left(X_{R},\varphi \left(x\right),r_{thr}\right)=\frac{1}{\left|R\right|}\sum_{i=1}^{\left|R\right|}\oint_{x\in \langle x_{i}|r_{thr}\rangle }\varphi \left(x\right)dx\;\;\;\;x_{i}\in X_{R},$$

where φ(x) is the known scenario’s threat Probability Density Function (PDF), and $r_{thr}$ is the threatened radius of drones. Note that $\langle x_{i}|r_{thr}\rangle$ is the circular region with center $x_{i}$ and radius $r_{thr}$. The threat value of each UAV can be obtained by integrating the threat PDF in the corresponding area. When location $x_{o}$ is outside the defined area D, we define $\varphi \left(x_{o}\right)$ equals the average value of threat PDF in D, i.e., $\varphi \left(x_{o}\right)=\frac{\sum \varphi \left(x\right)}{\left|D\right|},\forall x\in D,x_{o}\notin D$.

#### 2.2.3. Problem Construction

Network connectivity is the guarantee of information interaction between UAVs. Drones can share data with each other only when they are connected to the FANET. The ACP also needs a connected network to control and adjust drone swarms. In addition, ensuring the safety of UAVs is a prerequisite for the regular operation of FANETs. Today, all kinds of jamming and hijacking functions are integrated into various mobile anti-reconnaissance equipment, threatening UAVs’ security. Only when the safety of UAVs is guaranteed can drones cooperate to perform complex tasks.

Considering the above two aspects, we define the overall performance function f as a weighted sum of network connectivity and FANET threat metric as shown in Equation (4),

(4) $$\begin{array}{ll} f\left(X_{V},{\left\{\varepsilon_{m}\right\}}_{m\in M},\varphi \left(x\right),r_{thr},\rho \right)= & w^{C}f^{C}\left(X_{V},\rho \right)\\ & +w^{T}f^{T}\left(X_{R},\varphi \left(x\right),r_{thr}\right), \end{array}$$

where $w^{C}$ and $w^{T}$ are the weights for network connectivity and FANET threat metric, respectively. Then, we can formulate the problem in the following way so that it can be solved by the TA&CRFO algorithm:

(5) $$\underset{\begin{array}{l} x_{u}\in D,\;\;\;\;\forall u\in V\\ \varepsilon_{m}\subseteq V,\;\;\;\;\forall m\in M \end{array}}{\mathrm{maximize}}\;\;\;\;f\left(X_{V},{\left\{\varepsilon_{m}\right\}}_{m\in M},\varphi \left(x\right),r_{thr},\rho \right),$$

(6a) $$s.t.\;\;\;\;\left\|x_{i}-x_{j}\right\|\leq r^{C}\;\;\;\;\forall \left(i,j\right)\in \rho \left(m\right)$$

(6b) $$\left\|x_{i}-x_{j}\right\|\geq r^{S}\;\;\;\;\forall \left(i,j\right)\in V,\;\;\;\;u\neq v$$

(6c) $$\varepsilon_{m}\neq \varepsilon_{n}\;\;\;\;\forall \varepsilon_{m}\in M,\forall \varepsilon_{n}\in M$$

(6d) $$\left|\varepsilon_{m}\right|\geq 1\;\;\;\;\forall \varepsilon_{m}\in M$$

(6e) $$w^{C}>0,\;\;\;\;w^{T}<0$$

(6f) $$\varphi \left(x_{o}\right)=\frac{\sum \varphi \left(x\right)}{\left|D\right|},\forall x\in D,x_{o}\notin D$$

where $r^{C}$, $r^{S}$, and $r_{thr}$ represent the maximum allowable link length for reliable direct communication between two nodes, the minimum safety distance to avoid collisions between UAVs, and the threat radius of each relay UAV that can be deployed in a given three-dimensional Euclidean space, $D\in {\mathbb{R}}^{3}$, respectively. Constraint (6a) guarantees the UAVs’ reliable end-to-end communication. A safe flight distance is produced by constraint (6b) to reduce the danger of UAV crashes. Each UAV can only undertake one mission at a time and at least one UAV is required to complete each task, according to constraints (6c) and (6d). Constraint (6e) means that the overall performance function f increases as the FANET’s network connectivity increases and decreases as the FANET threat metric increases. Constraint (6f) describes the threat PDF definition for locations outside the simulation area.

## 3. Algorithm Description

By taking into consideration the mobility of the monitored targets and the dynamic changes at the scene, we present a description of how to build and run a continuous reconnaissance FANET under problem (5).

In order to facilitate readers’ understanding, we first introduce the principle of the basic PSO algorithm. Then, based on the PSO algorithm, we develop the TARFC. Considering the MUs’ movement toward the reconnaissance targets and the dynamic change of threat information in real scenarios, the TARFC has difficulty meeting real-time requirements. So, inspired by graph edit distance [31], we design the TED to measure the changes in network topology and scenario’s threat distribution at different times. Finally, combined with those mentioned above, we develop the TA&CRFO algorithm. This algorithm realizes the dynamic continuous reconnaissance goal of the FANET in a low-complexity way.

### 3.1. Rudimentary PSO Algorithm

PSO is a heuristic search algorithm proposed by J. Kennedy and R. Eberhart [32] in 1995. It is a random search algorithm that simulates biological activities and swarms intelligence in nature. The core idea is to use the information sharing of individuals in the group to guide the group’s movement in the problem-solving space. In the process of evolution from disorder to order, a feasible solution to the problem will be obtained. In addition to considering the group activities of simulated organisms, it is a swarm intelligence algorithm integrating individual cognition and social influence. 

Each particle in PSO iterates to improve its location in the simulation space of the optimization problem, selecting the best position thus far as the final solution at the end of the iteration.

For ease of understanding, we will use the following optimization problem to explain the PSO algorithm:

(7) $$\underset{x\in {\mathbb{R}}^{n}}{\mathrm{minimize}}\;\;\;\;h\left(x\right),$$

where $h\left(\cdot \right):{\mathbb{R}}^{n}\mapsto \mathbb{R}$ is called an objective function, ${\mathbb{R}}^{n}$ is the simulation space, and x is the decision variable.

We suppose that the PSO algorithm operates on a swarm of particles, each of which is represented by its position and velocity, i.e., $\left(x_{i},v_{i}\right)\in \mathbb{N}$. Each particle’s position corresponds to one of the potential solutions to the problem, as was previously mentioned. So, two special parameters emerge: pBest and gBest. The position that is the ith particle’s pBest, represented by $p_{i}$, is the best position that the particle has ever achieved. Analogously, the best position among the pBest of all particles is the unique gBest, represented by g. The ith particle’s velocity is updated by adding the stochastically weighted differences between its current position and both its pBest and the gBest, i.e.,

(8) $$v_{i}^{l+1}=wv_{i}^{l}+c_{1}u_{1}\circ \left(p_{i}^{l}-x_{i}^{l}\right)+c_{2}u_{2}\circ \left(g^{l}-x_{i}^{l}\right),\forall \left(x_{i},v_{i}\right)\in \mathbb{N},$$

where w is the inertia weight, $c_{1}$ and $c_{2}$ are individual cognition parameter and social influence parameter, the index l indicates the PSO algorithm’s lth iteration, both $u_{1}$ and $u_{2}$ are independent random vectors on ${\left[0,1\right]}^{n}$ with uniform distribution, and the superscript n represents the dimension of the simulation space.

In Equation (8), the inertia term controls the velocity’s amplitude, while the cognitive and social terms strike a balance between local search and global search. Equation (9), i.e.,

(9) $$x_{i}^{l+1}=x_{i}^{l+1}+v_{i}^{l+1},\forall \left(x_{i},v_{i}\right)\in \mathbb{N},$$

can be used to determine the new particle position based on the updated velocity.

**Figure 3:**
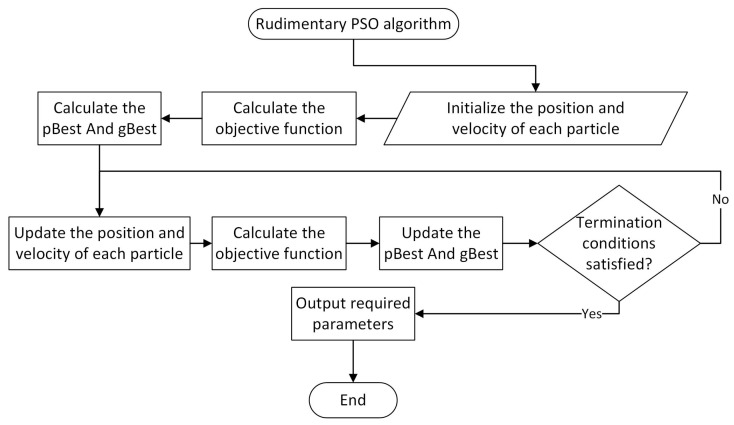
Flow chart of rudimentary PSO algorithm.

The positions of all the particles are updated iteratively in the simulation space by combining their velocities, which are modified in each iteration in accordance with Equation (8). The number of iterations, the swarm’s pace of convergence, the algorithm’s duration, and other factors can be used as termination conditions. The gBest becomes the top current solution to the unconstrained optimization problem (7) after the PSO algorithm terminates. Figure 3 shows the whole process of the rudimentary PSO algorithm.

Specifically, for FANETs, each particle in the PSO algorithm represents a complete FANET topology distribution scheme. The particle contains information about the UAVs’ position vector, the UAVs’ velocity vector, the FANET’s connectivity value, each UAV’s threat value, and the total performance of the scheme. During each iteration, the position vector of UAVs is updated based on the UAVs’ velocity vector in the previous round. The total performance values of all particles are compared, and the following iteration will be performed based on the particle with the best total performance.

### 3.2. PSO-Based TARFC

The PSO algorithm is suitable for solving unconstrained optimization problems such as Equation (7). Hence, in order to use our PSO-based TARFC algorithm, the Lagrange Multiplier and Karush–Kuhn–Tucker (KKT) methods [33] are employed to transform the constrained problem (5) into an unconstrained one. Thus, the reformulated problem is defined as

(10) $$\underset{\begin{array}{l} \left(x_{i},v_{i}\right)\in \mathbb{N},\forall x_{i}\in V\\ \varepsilon_{m}\subset V,\forall m\in M \end{array}}{\mathrm{maximize}}\tilde{f}\left(X_{V},{\left\{\varepsilon_{m}\right\}}_{m\in M},\varphi \left(x\right),r_{thr},\rho \right)$$

As shown in Equation (11), $\tilde{f}$ is then obtained by converting the constraints into penalty terms and adding them to the objective function:

(11) $$\begin{array}{l} \tilde{f}\left(X_{V},{\left\{\varepsilon_{m}\right\}}_{m\in M},\varphi \left(x\right),r_{thr},\rho \right)\\ =f\left(X_{V},{\left\{\varepsilon_{m}\right\}}_{m\in M},\varphi \left(x\right),r_{thr},\rho \right)\\ +\sum_{m\in M}\zeta_{m}{\left({\left[\underset{k=1,\ldots ,\left|\rho_{m}\right|-1}{\max}\delta \left(\rho_{m}^{k},\rho_{m}^{k+1}\right)-r^{C}\right]}^{+}\right)}^{2}\\ +\xi {\left({\left[r^{S}-\underset{\begin{array}{c} u,v\in M\cup R\\ u\neq v \end{array}}{\min}\delta \left(u,v\right)\right]}^{+}\right)}^{2} \end{array}$$

Note that $\zeta_{m}$ and ς are negative penalty coefficients corresponding to the end-to-end communication constraint and the safety requirement, respectively. ${\left[•\right]}^{+}$ stands for max{0,•}, which means that if the communication distance and safety distance between two UAVs meet the corresponding constraints, then the penalty term takes the value of zero. The particle i’s individual pBest and the gBest are defined as $x_{i}^{*}$ and $x^{*}$, and the solution $x^{*}$ that maximizes the $\tilde{f}$ would be the answer for the problem (5).

Algorithm 1 describes the PSO-based TARFC algorithm in full. Lines 1–4 are the preliminary stage. We first establish some fundamental characteristics (line 1). Then, we set all particles’ velocities to zero and their positions to random numbers evenly distributed over the defined simulation space. After that, we initialize all particles whose positions are to be random numbers uniformly distributed in the defined space of the problem (10) and whose velocities are to be all zeros. When the first time the particle i is initialized, its position automatically becomes its pBest position $x_{i}^{*}$, and the routing function ${\left\{\rho_{m}\right\}}_{i}^{*}$ determines its corresponding routing paths (lines 2–4). Following initialization, we identify the particle with the highest penalized performance metric value (since we defined $\zeta_{m}$ and ς are negative penalty coefficients). Then, change the particle g’s pBest position $x_{g}^{*}$ to the gBest position $x^{*}$, and its corresponding routing paths ${\left\{\rho_{m}\right\}}_{g}^{*}$ are also changed as the gBest position’s routing path ${\left\{\rho_{m}\right\}}^{*}$.

In the iterative stage (lines 5–19), we first obtain the current location of the ACP and mission-execution UAVs, $X_{A}$ and $X_{M}$, as well as the latest threat PDF, φ(x), in the scenario. Guided by the above, relay UAVs’ velocity, position, routing, and other attributes are updated periodically. To be more specific, the particles’ position and speed are changed stochastically based on both their unique pBest position and the overall gBest position as

(12) $$\left[\begin{array}{c} x^{l+1}\\ v^{l+1} \end{array}\right]=\left[\begin{array}{cc} 1-c_{1}u_{1}-c_{2}u_{2} & w\\ -c_{1}u_{1}-c_{2}u_{2} & w \end{array}\right]\left[\begin{array}{c} x^{l}\\ v^{l} \end{array}\right]+I_{2}\left[c_{1}u_{1}+c_{2}u_{2}\right]\left[\begin{array}{c} p^{l}\\ g^{l} \end{array}\right]$$

The index l in Equation (12) is the algorithm’s lth iteration. Both $u_{1}$ and $u_{2}$ are uniformly distributed random vectors.

It should be noted that during the simulation, the speed change could be out of the actual. Hence, we bring in a velocity clamping method [34] as shown in Equation (13),

(13) $$v_{i,j}^{l+1}=\{\begin{array}{cc} v_{i,j}^{l+1} & \mathrm{if}\;v_{i,j}^{l+1}\in \left[-V_{j}^{\max},V_{j}^{\max}\right]\\ \frac{v_{i,j}^{l+1}}{\left|v_{i,j}^{l+1}\right|}V_{j}^{\max} & otherwise \end{array}$$

where $v_{i,j}^{l+1}$ is the jth element of $v_{i}^{l+1}$, and $V_{j}^{\max}$ is the velocity clamping threshold. If the modified position can obtain better results in $\tilde{f}$, the pBest and gBest for each particle will be altered, as explained in lines 11–18. Eventually, the algorithm terminates when it meets the termination condition, i.e., it runs to a preset number of iterations, or the results no longer improve in a certain number of iterations (line 19).
**Algorithm 1.** Threat Avoidance and Reconnaissance FANET Construction
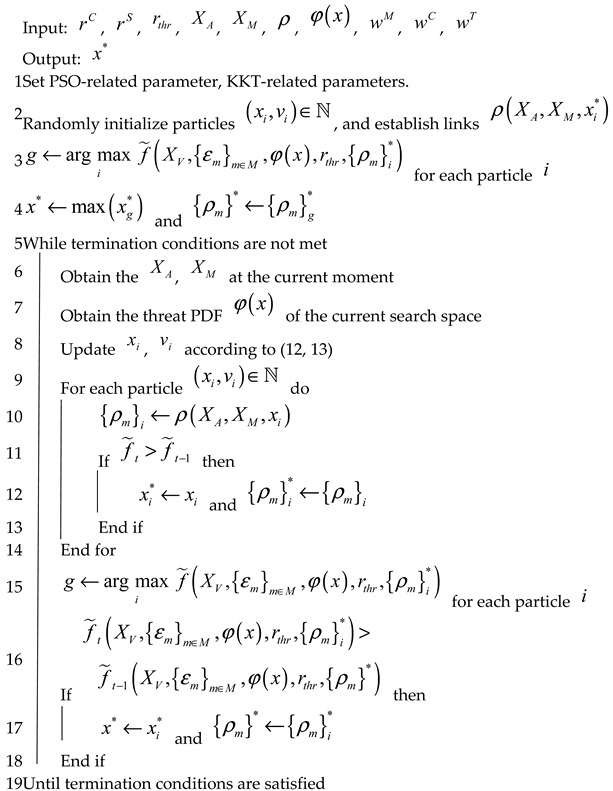


Although the TARFC algorithm provides FANETs with a feasible network construction method to perform reconnaissance tasks in threatening scenarios, in most real reconnaissance missions, such as military target reconnaissance, to remain undetected, UAVs cannot influence the movement of the detected targets or the relevant scene’s alteration. This puts forward a “sustainable” reconnaissance requirement for the FANET. As for algorithm II, every FANET adjustment according to the changing targets or scene requires a large number of iterations, which is extremely time consuming. For this reason, in the following Section 3.3 and Section 3.4, we first propose an indicator called Total edit distance to measure the variation degree of the FANET and the threat PDF change in related scenarios. Secondly, a low-complexity algorithm named TA&CRFO is proposed. According to the above indicators, the algorithm can conduct two different adjustment modes for the FANET to meet the needs of “sustainable” in real situations.

### 3.3. Total Edit Distance

Inspired by the graph edit distance [31,35], we innovate the Total Edit Distance concept to measure the changes in the FANET and the extent of changes in the threat PDF. Our basic philosophy is to avoid frequent routing updates and iterations to reduce computational overhead and preserve overall efficiency to the greatest extent possible. To do this, we will judge whether to execute overall coordination by sending control instructions through ACP or let each node perform distributed adaptive adjustment according to the range of the TED.

Prior to introducing the TED, we briefly describe the graph edit distance [35] here to aid. In graph theory, the graph edit distance measures the dissimilarity between graph $\Omega_{1}$ and graph $\Omega_{2}$. Many graph editing operations, such as node and edge additions and deletions, can change one graph into another. With each graph edit operation’s cost, the cost of each operation is summed to obtain the total cost of converting the graph $\Omega_{1}$ to $\Omega_{2}$, and the smallest cost in this process is defined as the graph edit distance from $\Omega_{1}$ to $\Omega_{2}$. Note that different operations usually have various cost functions. Thus, two different sets of operations, with the same outcomes in altering the graph $\Omega_{1}$ to $\Omega_{2}$, may be of relatively large distinction regarding the total cost.

We first establish the FANET edit distance to measure the change in the FANET. Then, the threat edit distance is defined to record the fluctuation of threat PDF in associated areas, both of which are based on the graph edit distance principle. As a result, the FANET edit distance and the threat edit distance are added to form the TED.

Unlike normal graph edit operations, our system does not consider the addition or damage of UAV nodes. So, the node sets are the same at different times. That is to say, the FANET edit operations only contain edge changes, such as edge insertion, edge deletion, and edge length change.

We symbolize the FANET as a graph ϑ(t). The graph consists of the set of nodes N, the set of edges σ(t) and their corresponding positions set $P_{N}\left(t\right)$. To transform ϑ(t) to ϑ(τ), where t and τ are adjacent time with t<τ, the minimum edge insertions and deletions can be expressed as

(14) $$d_{1}(t,\tau )=\sigma \left(\tau \right)-\sigma \left(t\right),$$

(15) $$d_{2}\left(t,\tau \right)=\sigma \left(t\right)-\sigma \left(\tau \right),$$

where $d_{1}\left(t,\tau \right)$ is the edge insert operation between time t and τ, the $d_{2}\left(t,\tau \right)$ is the edge delete operation between time t and τ, respectively.

More importantly, we must pay special attention to the changes in edge lengths since the drones in the FANET move vigorously and frequently. Because the edge length between two nodes is closely related to their communication performance, the optimal routing path may differ from time t to time τ. Hence, the total amount of edge length changes between time steps t and τ is how we define the edge length change operation; that is,

(16) $$d_{3}\left(t,\tau \right)=\sum_{i,j\in N}\left|{\left\|p_{i}\left(t\right)-p_{j}\left(t\right)\right\|}_{2}-{\left\|p_{i}\left(\tau \right)-p_{j}\left(\tau \right)\right\|}_{2}\right|$$

where $p_{i}\left(t\right)$ stands for the position of node i in time step t. Note that TED is applicable to simulations of different dimensions. Depending on the simulated scene’s dimensionality, the node i’s position $p_{i}$ can be equivalent to a two-dimensional $\left(x_{i},y_{i}\right)$ vector, a three-dimensional $\left(x_{i},y_{i},z_{i}\right)$ vector, or even a higher-dimensional space vector.

We lastly define the threat edit distance to measure the changes in the scenario’s threat density distribution at different times. In the duration of a reconnaissance mission, obtaining global information is laborious and impractical. Moreover, each drone in the FANET is threatened by a limited area in our model. Hence, the threat edit distance is defined as the average change in each UAV’s threat value between time t and τ, i.e.,

(17) $$d_{4}\left(t,\tau \right)=\frac{1}{N}\sum_{i=1}^{\left|N\right|}\left|\oint_{p\in \langle p_{i}|r_{thr}\rangle }\varphi \left(p\left(t\right)\right)dx-\oint_{p\in \langle p_{i}|r_{thr}\rangle }\varphi \left(p\left(\tau \right)\right)dx\right|$$

Finally, the TED between time t and τ is defined as

(18) $$\theta \left(\vartheta \left(t\right),\vartheta \left(\tau \right),P\right)=\sum_{i=1}^{4}w_{i}\cdot d_{i}\left(t,\tau \right),$$

where P is the set of all active connections, and $w_{i}$ is the weight parameter of each edit operation $d_{i}$.

A large value of TED implies that the FANET has changed a lot, or the regional threat density distribution varies greatly. In such cases, centralized scheduling is needed to tune the FANET. If the value of TED is within a reasonable range, we may enable each UAV to adjust its speed adaptively to optimize reconnaissance and communication performances. So, the TA&CRFO algorithm is developed to achieve dynamic, persistent surveillance in a less complex way.

### 3.4. TA&CRFO

We now go into depth about our TA&CRFO algorithm in this subsection. Algorithm 2 presents the pseudo-code. In the preparatory phase (lines 1–2), which usually is the beginning of reconnaissance missions, the high-altitude UAV, known as ACP, constructs the initial FANET by Alg. 1 based on available information. Then, each low-altitude UAV flies to the position specified by the high-altitude UAV. When they arrive, we abstract the FANET into a graph and define it as a reference graph $\vartheta_{ref}$ for a particular time.

During the iterative stage (lines 3–16), the high-altitude UAV collects the position information of the scouted targets and evaluates the fluctuation of threat density distribution in the scenarios. Meanwhile, in time t, the ACP calculates the TED $\theta \left(\vartheta_{ref},\vartheta \left(t-1\right),P\right)$ between reference graph $\vartheta_{ref}$ and the FANET graph at time step t−1 in the current scenario. The $\theta \left(\vartheta_{ref},\vartheta \left(t-1\right),P\right)$ value represents how much the FANET and threat PDF in the scenario have changed from the reference time. We assert that the cumulative changes are insignificant if the TED between them is below the threshold λ. Therefore, by Equations (19) and (20), we have each low-altitude drone adaptively alter its position (lines 7–11).

The right side of Equation (19) is the gradient of the total performance function concerning the position of node $x_{i}$ at time t. With neighbor UAVs’ position information and the threat PDF of related areas, each node’s gradient value can be easily obtained.

On the other hand, if the TED is greater than λ, we believe that the distributed position adjustment has lost its meaning since the routing path or scenario’s threat PDF may have altered too much. Therefore, we use Algorithm 1 again to reconstruct the FANET network in a centralized way and set the newly configured network as the reference graph $\vartheta_{ref}$.
**Algorithm 2.** Dynamic Threat Avoidance and Continuous Reconnaissance FANET Operation
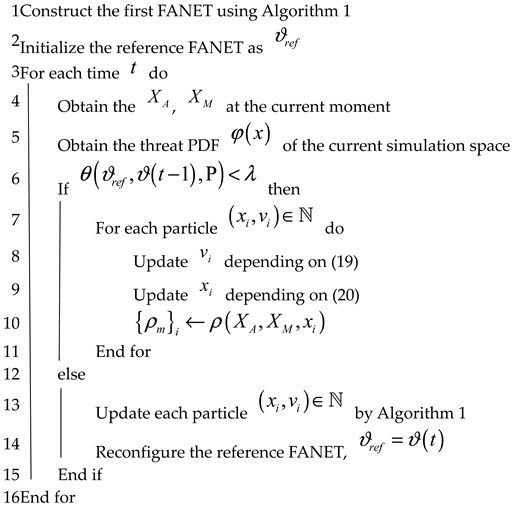

(19) $$v_{i}\left(t\right)=\nabla_{x_{i}}f\left(t\right)=\nabla_{x_{i}}f\left(X_{V}\left(t\right),{\left\{\varepsilon_{m}\right\}}_{m\in M},\varphi \left(x\left(t\right)\right),r_{thr},\rho \right)$$

(20) $$x_{i}\left(t+1\right)=x_{i}\left(t\right)+v_{i}\left(t\right)$$

In other words, at each time, the high-altitude UAV will decide whether to issue instructions to all low-altitude drones for overall control according to $\theta \left(\vartheta_{ref},\vartheta \left(t-1\right),P\right)$. If low-altitude drones receive those instructions, they will obey them. Otherwise, they continue their adaptive location optimization method using local information.

## 4. Results

This article considers a 3D scene with a randomly generated threat PDF, in which multiple monitoring UAVs perform reconnaissance tasks at different locations. Various numbers of relay UAVs are provided to forward the detected targets’ relevant data and the variation in threat PDF at the scene. The high-altitude UAV, known as ACP, oversees the entire FANET in the whole process.

For the sake of effective comparison, four algorithms, RWP [36], PSO, TARFC, and TA&CRFO, are respectively implemented under the condition that the values of all parameters are consistent. The simulation process is executed on MATLAB. In the simulation, we assume that there are no projectiles in the environment, such as birds or obstacles, which may block the flight path of the UAV, and it is assumed that the battery capacity of the UAVs meets the requirement of continuous reconnaissance.

The alteration of each index in the process of algorithm execution is compared, and the reasons for different results obtained by different algorithms are analyzed. Simulation results demonstrate that the FANET net constructed by the TA&CRFO algorithm is 6.06~7.23% lower than TARFC in connectivity and 0.46~1.21% higher in UAV’s average threat value, but the time consumption of the algorithm is only 19.86~20.31% of TARFC. At the cost of other performances’ slight impairment, the TA&CRFO achieves UAV’s limited distributed control and a significant reduction in computing overhead.

Table 3 describes the simulation scenario and lists the parameters during the simulation. The horizontal dimension of the simulated scenario is set as 1 km × 1 km, and the flight height of the UAV is set to 100 m and 200 m. In valid experiments, the number of RUs ranged from 7 to 19. Given that too few RUs cannot establish communication links at multiple reconnaissance sites simultaneously, too many RUs may not offset the outlay despite the increased performances. We assume that the relay drones can either hover or travel at a maximum speed of 15 m per second. The shortest path routing algorithm, whose link usage is calculated as the cube of its length, is the default routing protocol. In terms of distance, the low-altitude UAVs’ safety distance, maximum communication distance, and the diameter of the perceived threat area are set to 20 m, 200 m, and 50 m, respectively. Our work not only uses the original PSO algorithm as the comparison algorithm but also constructs a new one based on the PSO algorithm. Therefore, the PSO-related parameters are listed explicitly for ease of reference. Finally, the parameters related to the KKT method and our algorithms are also listed in Table 3.

### 4.1. Scenario Exhibition

**Figure 4:**
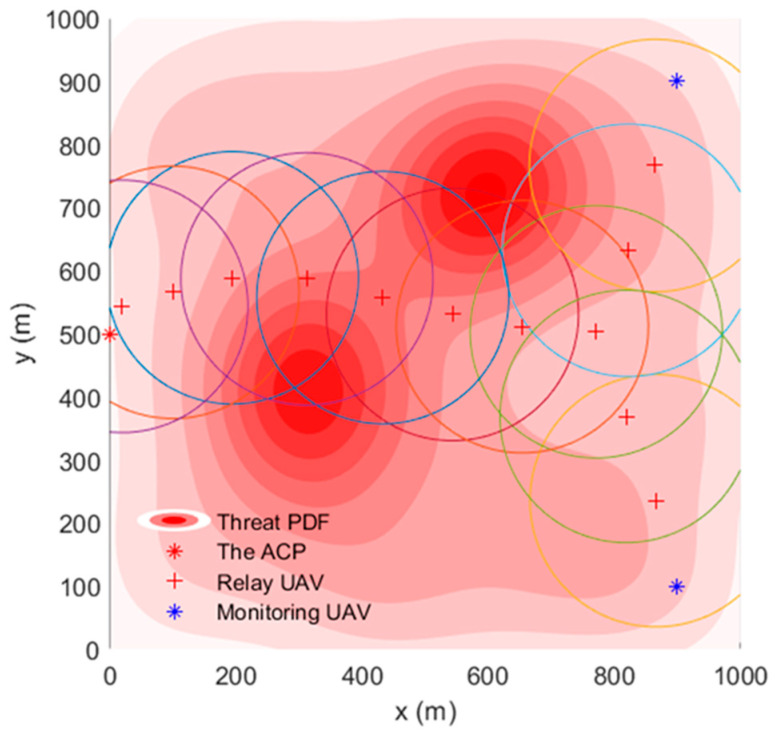
UAVs’ topology using TA&CRFO algorithm at *t_i_*.

In the FANET consisting of the ACP, 12 relay UAVs, and two monitoring UAVs, the TA&CRFO algorithm is used to simultaneously carry out continuous reconnaissance of mobile targets. Figure 4 shows the simulation results at $t_{i}$. The circle with the relay UAV as the center in Figure 4 represents the maximum communication range of the RUs. During the reconnaissance, the monitoring UAVs move closely with the movement of monitored targets. Since the trajectory of targets is unpredictable for the ACP, the MUs’ movements are completely controlled by themselves. The RUs adjust their positions according to the displacement of the MUs and the variation in threat PDF to avoid hazards and ensure the communication quality between the ACP and the MUs.

### 4.2. The Simulation Trajectory

**Figure 5:**
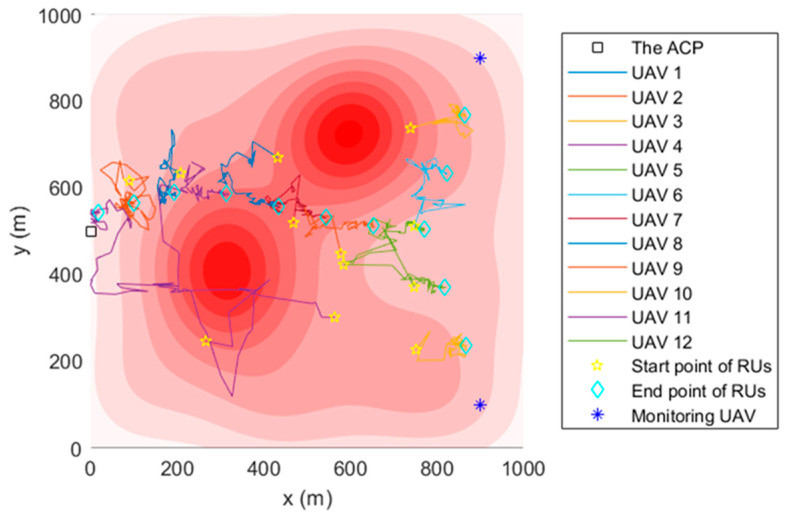
Trajectory of the FANET in a certain time period (Top view).

**Figure 6:**
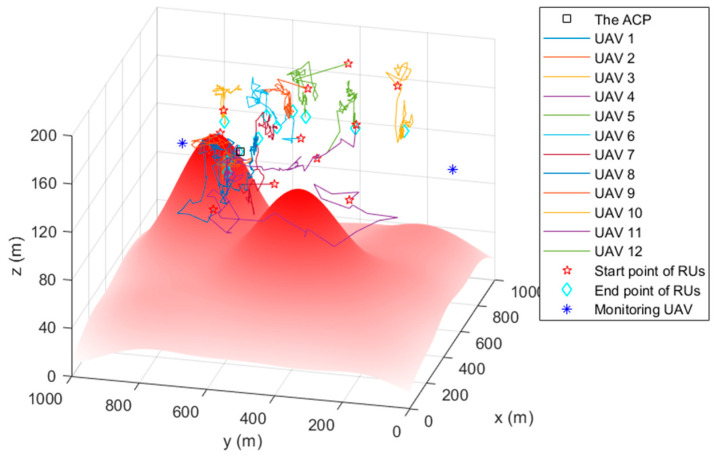
Trajectory of the FANET in a certain time period (Left view).

**Figure 7:**
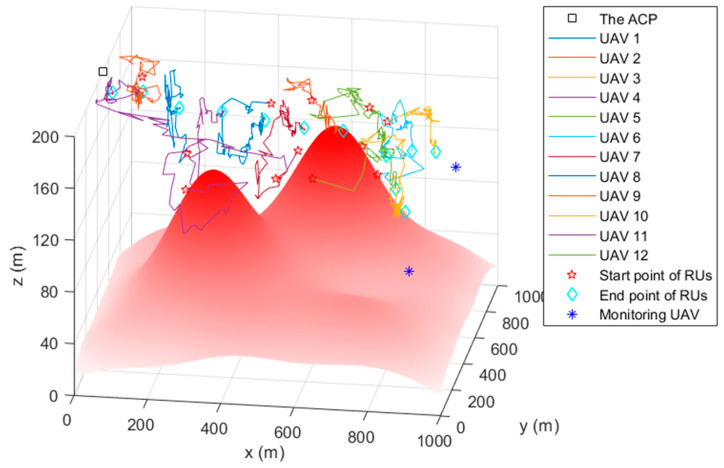
Trajectory of the FANET in a certain time period (Front view).

Figure 5, Figure 6 and Figure 7 are the trajectory diagrams of each UAV in the continuous reconnaissance process using TA&CRFO. Only a period of FANET trajectory is shown to facilitate readers’ identification. Since the whole network is in 3D space, a multi-angle display is necessary to clearly show the changes in the FANET in the continuous reconnaissance process. Thus, Figure 5, Figure 6 and Figure 7 are presented as the process’s top, left, and front views. Inside these pictures, the colors and shapes of the markings represent different types of UAVs. The lines in different colors represent the moving track of each relay UAV during this time period. Through those figures, it can be observed that the newly formed FANET can play a better relay role in monitoring UAVs at (900, 100, and 100) m and (900, 900, and 100) m, and keep away from positions in high-threat areas of the moment.

### 4.3. Connectivity

**Figure 8:**
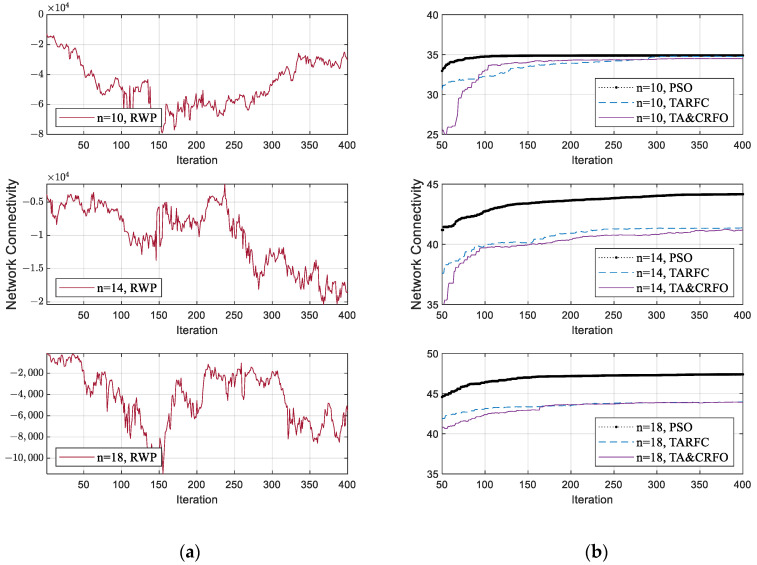
The Network Connectivity fluctuation of algorithms in the iterative process of FANET construction: (**a**) The fluctuation of the RWP algorithm; (**b**) The fluctuation of other algorithms.

Figure 8a,b show each algorithm’s network connectivity in the iterative process of FANET construction. To facilitate the demonstration, we select the FANET construction process of different algorithms in the same scene, including identical threat PDF and the movement of monitored targets. Due to limited space, only the process where the RUs’ number is 10, 14, and 18 is displayed, respectively. The RWP algorithm is listed separately in Figure 8, considering that the value range of this algorithm’s communication performance is quite different from others.

RWP is a random movement model. In this model, nodes move randomly without any constraints. The nodes are random in speed, direction of motion, and are independent of each other. However, our reconnaissance targets are not entirely random in the real world. Therefore, after the distance constraint in Equation (6) is converted into a penalty coefficient (Equation (11)), the increase in the penalty term makes the network connectivity of RWP fluctuate randomly in a wide range (shown in Figure 8a). Moreover, the network connectivity of the FANET constructed by RWP does not converge with the iterative process since the nodes’ movement in the RWP algorithm has characteristics of randomness and irregularity.

Figure 8b shows the convergence process of PSO, TARFC, and TA&CRFO’s network connectivity. Among them, PSO only focuses on optimizing network connectivity, while TARFC and TA&CRFO also consider threat avoidance during the continuous reconnaissance process. Therefore, PSO is slightly better than TARFC and TA&CRFO in terms of network connectivity. TA&CRFO is a simplified version of TARFC in terms of complexity, but as can be seen from the chart, the performance of network connectivity is comparable to that of TARFC. In addition, as the RU number increases, the network connectivity of those algorithms also increases, and their performance shows a tendency toward convergence.

### 4.4. FANET Threat Metric

**Figure 9:**
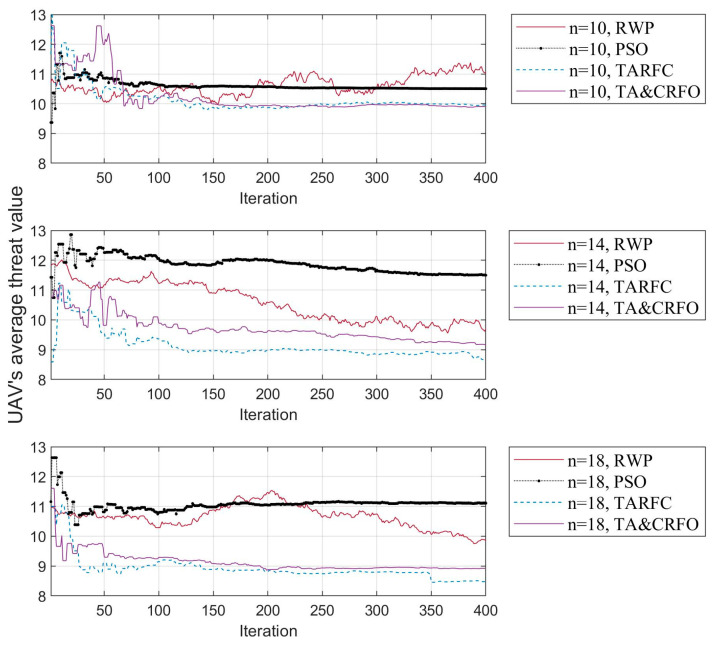
The UAV’s average threat fluctuation of different algorithms in the iterative process of FANET construction.

In Figure 9, the fluctuation of the UAV’s average threat value is presented. Four different colored curves in the picture represent four different algorithms. Similarly, the FANET threat metric with 10, 14, and 18 RUs in the network illustrates the trend of UAV’s average threat value with the number of RUs.

As mentioned above, the excessive randomness of RWP makes the FANET threat metric fluctuate randomly within a wide range of values and does not tend to converge in the iteration process. On the contrary, other algorithms gradually find the UAVs’ optimal position in the iteration process, and their FANET threat metric can converge to a small range. In addition, by longitudinal comparison of the three subgraphs, the spatial freedom of each relay UAV increases as the number of RUs increases, which allows them to optimize themselves by reaching better relay positions.

### 4.5. Detailed Comparison of Algorithms

In this section, we examine the four algorithms from a variety of perspectives, including the overall performance, average threat value for UAVs, longest and shortest link distances between UAVs, and algorithm complexity. In order to ensure the effectiveness of comparative experiments, all parameters in different algorithm experiments are consistent. The results are averaged over 20 repeated experiments.

**Figure 10:**
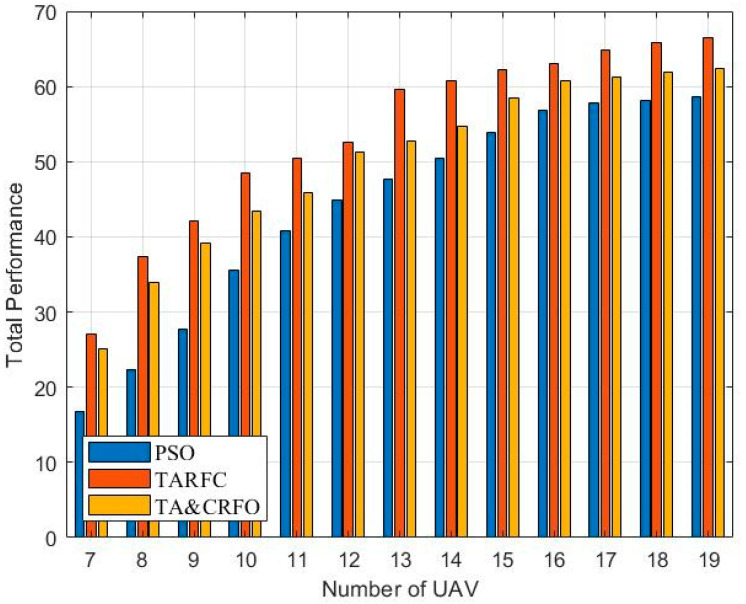
Total Performance of algorithms.

Due to the randomness of RWP, its total performance (Equation (11)) is not ideal and the value varies rapidly. Thus, Figure 10 shows the overall performance variations of PSO, TARFC, and TA&CRFO as the number of available RUs increases. We notice that the connectivity value of these three algorithms is between 34 and 50, after which they tend to be stable, while the FANET threat metric is in the range of 8–12. To ensure the fluctuation of these two values is consistent, the weight factors $w^{C}$ and $w^{T}$ were set to 0.5 and 2.5, respectively.

The network’s connectivity requires a certain number of UAVs as a guarantee, so the number of RUs starts from seven, according to the simulation experiment. It can be seen that with the increase in RUs, the total performance of the three algorithms becomes larger. However, it should be noted that the performance improvement of PSO mainly comes from network connectivity, so its growth trend gradually decreases. Whereas the performance of TARFC and TA&CRFO is first improved due to network connectivity; then, as the RUs’ option space increases, low-threat density areas are selected as relay positions.

**Figure 11:**
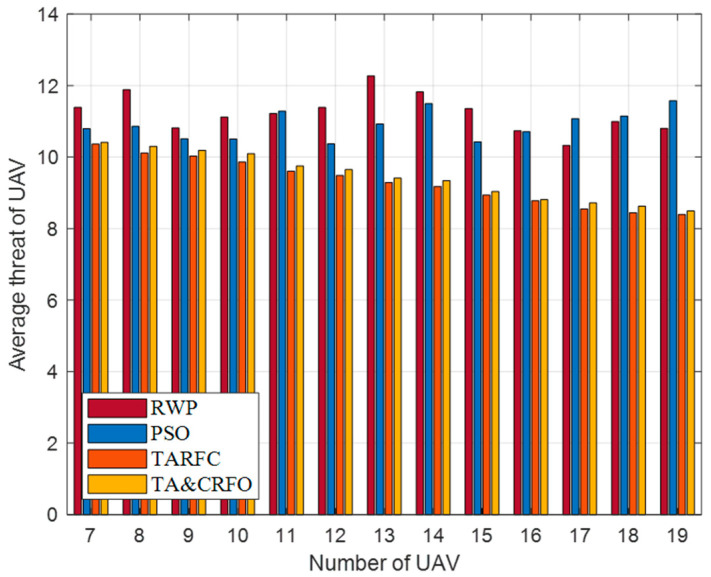
UAV’s average threat value with different algorithms.

Figure 11 presents the UAV’s average threat value obtained by constructing the FANET with different algorithms. It is worth mentioning that most places in the scene have a threat PDF between 6 and 13. It can be easily seen that the UAV’s average threat value in RWP and PSO does not decrease with the increase in RU number but fluctuates randomly. This is because RWP moves randomly in space, while PSO only cares about the interconnection between nodes and does not consider the threat PDF information in the scene. On the contrary, the UAV’s average threat value in TARFC and TA&CRFO is lower than that of the above two algorithms and gradually decreases with the increase in relays. Among them, TARFC is slightly better than TA&CRFO, mainly because TA&CRFO performs distributed adjustments sometimes and cannot keep the optimal global state at all times.

**Figure 12:**
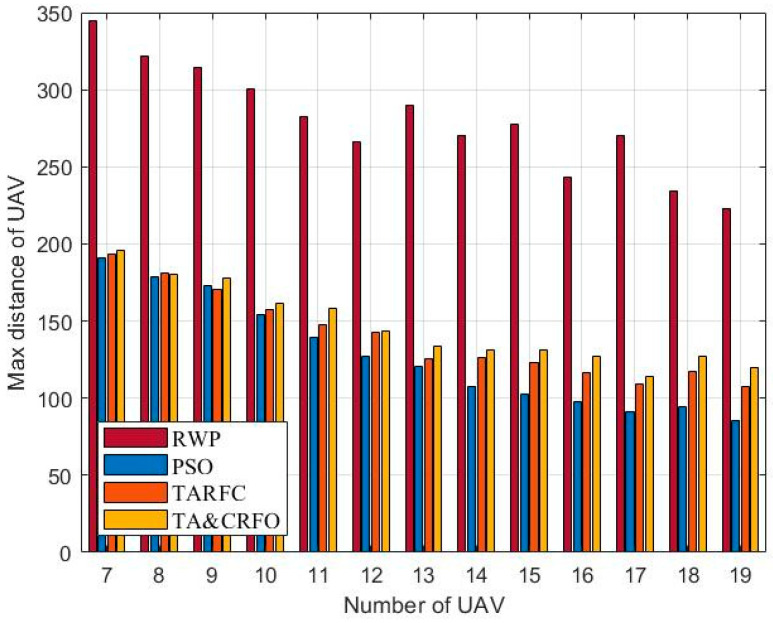
The longest link distance of FANET constructed by different algorithms.

**Figure 13:**
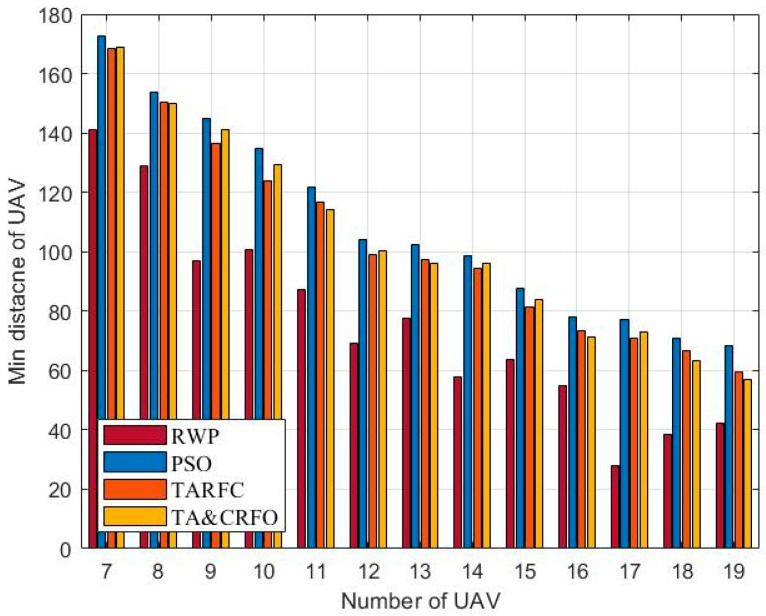
The shortest UAV distance of FANET constructed by different algorithms.

Figure 12 and Figure 13 show the longest and shortest UAV distance of the FANET constructed by different algorithms. Taken these two metrics together, they demonstrate FANET’s compactness and uniformity. As shown in the figures, other algorithms can meet the constraints of the maximum communication distance of 200 m between UAVs and the minimum safe distance of 50 m, except RWP. Among them, with the increase in relays, the decrease in PSO’s longest link distance is more significant than that of TARFC and TA&CRFO, while the decline of the shortest UAV distance is smaller than that of TARFC and TA&CRFO. It can be seen that PSO can make the FANET’s nodes tend to be evenly distributed. However, for realistic scenarios with uneven threat PDF, algorithms such as TARFC and TA&CRFO obviously have more advantages since they can bypass the high-risk area.

We assume that the high-altitude UAV control center has superior operational capability and can obtain the algorithm’s optimization results in milliseconds. However, the FANET results constructed by the algorithm cannot be provided in time due to the limitations of our simulation equipment. To visualize the algorithms’ complexity, we use the same parameter settings and scene settings to simulate two detected targets at different positions moving 300 m in a straight line. Continuous reconnaissance is carried out for this process, and the total execution time of each algorithm is calculated (target one from (900, 900, and 100) m to (660, 720, 100) m, and target two from (900, 100, and 100) m to (900, 400, and 100) m). It is assumed that only when the algorithm completes the FANET’s construction of time step t will the monitored target will arrive from the position at t to the position at τ.

**Figure 14:**
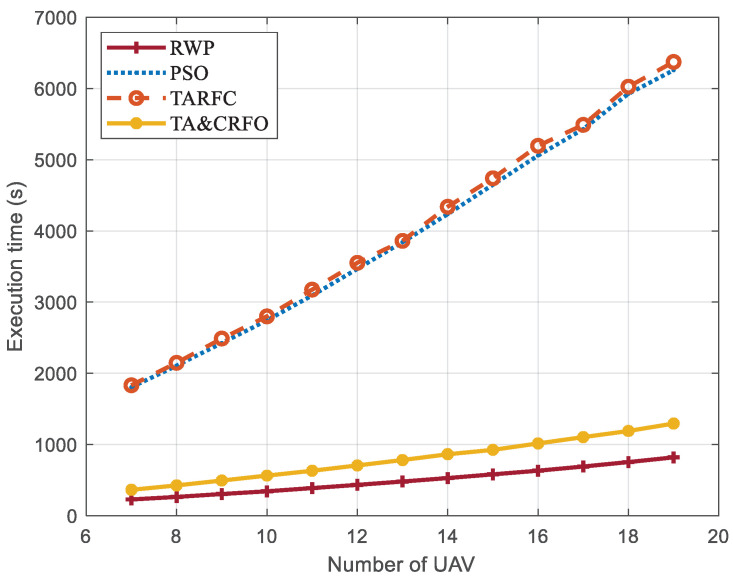
The average execution time of different algorithms.

Figure 14 shows the execution time of different algorithms to complete the entire continuous reconnaissance process. There is no iterative process in RWP, and the selection process at each moment is entirely random, so its average execution time is about 12.46~13.11% of that of PSO and TARFC. TARFC has a similar complexity as PSO, but it optimizes the maintenance of network connectivity and the avoidance of high-threat areas. Based on the original TARFC, TA&CRFO is designed to selectively realize the self-adaptive regulation of UAVs, which effectively reduces the iterative operation of the algorithm. When the parameter threshold λ is set to 5.23, the TA&CRFO’s execution time is about 19.86~20.31% of that of TARFC, even though its effect is slightly inferior to that of the TARFC algorithm in other aspects (Figure 9, Figure 10, Figure 11, Figure 12 and Figure 13).

## 5. Conclusions and Future Works

This paper presents a layered structure of FANET, in which high-altitude UAVs act as ACPs and multiple UAVs are used for remote relaying and data collection. A “sustainable” dynamic reconnaissance mechanism, TARFC, is constructed considering the movement of reconnaissance targets and the change in various adverse factors in the scenario.

During the simulation, we found that for the FANET, maintaining network connectivity and avoiding local threats during mission execution are two conflicting requirements. The basic PSO only considers network connectivity, which has the best performance in this aspect but is inferior to TARFC and TA&CRFO in terms of threat avoidance. For the latter two algorithms, the weighting values of network connectivity and threat avoidance during simulation must be carefully considered according to realistic requirements. The overall performance of the TA&CRFO algorithm is slightly lower than that of TARFC, but its computational overhead is effectively reduced by decreasing the iterative process. In addition, the computation time required by TA&CRFO increases more slowly as the number of UAVs used in the simulation increases. So, the TA&CRFO algorithm is more suitable for larger-scale FANET.

Of course, the design of indicator functions such as TED also determines the simulation results to a large extent and should be paid special attention.

In this work, the network construction process of Algorithm 1 is carried out on the high-altitude UAV, namely, ACP, which is a centralized approach. The TA&CRFO algorithm is semi-distributed since local neighbor information is used between UAVs when the TED is less than the threshold value. This approach reduces communication overhead and dependence on the central node ACP. In the future, a fully distributed continuous reconnaissance algorithm that completely abandons the central node will bring a greater leap in FANET’s adaptability and survivability. In addition, a complex sensing model and connectivity disruption caused by UAV failure will be further considered.

## Figures and Tables

**Table 1:** List of acronyms.

Acronym	Description
TA&CRFO	Dynamic threat avoidance and continuous reconnaissance FANET operation
UAV	Unmanned aerial vehicle
TARFC	Threat avoidance and reconnaissance FANET construction algorithm
FANET	Flying ad hoc network
ACP	Airborne command and control platform
LoS	Line-of-sight
PSO	Particle swarm optimization
TED	Total edit distance
AUDS	Anti-UAV defensive system
MUs	Monitoring UAVs
RUs	Relay UAVs
PDF	Probability density function
KKT	Karush–Kuhn–Tucker method

**Table 2:** List of variables.

Variable	Description
A	The ACP of FANET
U	Set of low-altitude drone swarms
R	Set of all RUs
M	Set of all MUs
$x_{i}$	Location of node i
V	Node Set
$X_{V}$	Set of locations of all nodes in V
$\varepsilon_{m}$	The MU assigned to mission m
ρ(m)	Routing path for mission m
Ρ	Set of all active links
f	Overall performance function
$f^{C}$	Network connectivity function
$f_{i,j}^{C}$	Wireless connectivity quality between i and j
$f^{T}$	FANET threat metric
$r_{thr}$	Threatened radius of UAV
φ(x)	Threat PDF in related areas
$r^{C}$	Maximum communication radius between UAV
$r^{S}$	Minimum safety radius between UAV
t, τ	Time
ϑ(t)	FANET in time t
N	Node set in graph theory model
σ(t)	Edge set in graph theory model
$P_{N}\left(t\right)$	Nodes’ position set in graph theory model

**Table 3:** Setting of simulation parameters.

Parameter	Value
Scenario	
Horizontal dimensions	1000 × 1000 m
Vertical dimension	[100~200] m
Number of RUs	[7~19]
RUs’ speed	[0~15] m/s
Distances	
Minimum safe distance, $r^{S}$	20 m
Maximum link distance, $r^{C}$	200 m
UAV’s threat perception radius, $r_{thr}$	25 m
PSO-related	
Number of particles, $N_{p}$	50
Number of iterations	400
Inertia weight, w	0.7192
Cognitive parameter, $c_{1}$	1.4472
Social parameter, $c_{1}$	1.4472
KKT and FANET-related	
ξ $,\;\zeta_{m}$	0.5
Connectivity weight, $w^{C}$	0.5
Weight of FANET threat metric, $w^{T}$	2.5
Weight, of edit operations, $w_{1},w_{2},w_{3},w_{4}$	2, 2, 0.3, 40
TED’s threshold parameter, λ	5.23
